# Why epigenetics is (not) a biosocial science and why that matters

**DOI:** 10.1186/s13148-022-01366-9

**Published:** 2022-11-11

**Authors:** Luca Chiapperino, Francesco Paneni

**Affiliations:** 1 STS Lab, Institute of Social Sciences, Faculty of Social and Political Sciences, University of Lausanne, 1015 Lausanne, Switzerland; 2 Quartier UNIL-Mouline, Institute of Social Sciences, Bâtiment Géopolis, Bureau 5556, 1015 Lausanne, Switzerland; 3 University Heart Center, Department of Cardiology, University Hospital Zurich, Zurich, Switzerland; 4 Center for Translational and Experimental Cardiology (CTEC), Department of Cardiology, University Hospital Zurich and University of Zurich, Zurich, Switzerland

**Keywords:** Epigenetics, Cardio-epigenetics, Post-genomics, Biosocial, Science and technology studies, Interdisciplinarity

## Abstract

Epigenetic modifications offer compelling evidence of the environmental etiology of complex diseases. Social and biographical conditions, as well as material exposures, all modulate our biology with consequences for risk predispositions and health conditions. Elucidating these complex *biosocial loops* is one of the main challenges animating epigenetics. Yet, research on the development of epigenetic biomarkers often pulls in a direction that departs from a view of biological determinants of health embedded in their social and material environment. Taking the example of the epigenetics of cardiovascular diseases, this paper illustrates how common understandings of epigenetic biomarkers strongly lean toward considering them as mere targets for molecular intervention, rather than as correlates of a complex biological and social patterning of disease. This reductionism about biosocial dynamics of disease, we argue, hampers the pursuit of the goals epigenetics *has given itself* (in cardiology and beyond). If epigenetic mechanisms point to the deep socio-environmental embeddedness of our health, we conclude, future designs and methods of this research may require an improved methodological consideration of a biosocial perspective.

## Introduction

Epigenetic modifications, such as DNA methylation, histone modifications, and non-coding RNAs, offer a precious insight into the biological consequences of social conditions, material exposures, and biographical events [1]. Questions about the environment in medicine have a far-reaching history: In fact, one could argue, epigenetic research only re-actualizes central tenets of the long history of biology and medicine [2]. However, the specificity of epigenetics’ revival of such environmental thinking in medicine is that it approaches these questions through the tools, techniques, and ways of doing science inaugurated with genomics [3]. And this is, often, also a source of dissonance for many observers.

In fact, genome sequencing technologies developed between the 1980s and the early 2000s were designed for a different purpose: to elucidate the *genetic* basis of diseases. As both historians of science [3] and life scientists [4] have argued, they were neither meant to nor fit well with the purpose of disentangling complex interactions and looping effects among biological and environmental factors. These tools rather got repurposed into multi-omics and big data analytics when it became evident they could not deliver on the promise of explaining complex diseases solely on genomic grounds [5, 6]. Yet, this move was not accompanied by complementary attention paid to precise and accurate measurements of the environment. Post-genomic ways to measure the interplay of genetic and environmental factors in complex diseases “are entangled in ways that greatly obscure insight,” as recently argued by Science editor-in-chief Jeremy Berg [4, p. 15]. Let us take, as an example, cardiovascular diseases. It is undeniable that the clinical value of genomics for cardiovascular conditions has grown substantially over the last decade. Polygenic risk scores and rare variants make genomics and its tools increasingly relevant to cardiological care, although to differing degrees depending on the condition [7]. Yet, researchers at the dawn of the twenty-first century face an altogether different set of challenges to explain these conditions. What does the genetic heterogeneity of cardiovascular phenotypes mean for disease etiology? How to explain those effects that cannot be reduced to genetic factors alone? And what about nongenetic factors in these processes?

More than offering us a privileged view of the genetic origins of complex diseases, the tools and questions of genomics have progressively “undermined their core driving concept, the concept of the gene” [8, p. 5]. Today, omics methods find a prolific usage beyond the elucidation of the genetic drivers of complex diseases. First, the life sciences community increasingly recognizes the intrinsic biases in estimates of genotype–phenotype associations. Genetic associations can overlook the non-genetic causal path leading to these associations: For instance, phenomena such as population stratification or dynastic effects^1^ inflate genetic associations and causality in genotype–phenotype association studies [9]. In other words, statistical associations between genotypes and phenotypes should take population phenomena into account, especially in the case of complex traits that depend more on social structures and environmental factors than on heritable traits [9].

Second, fields like epigenetics remind us that the biological processes leading to disease often cannot be separated from their environments. In epigenetics, social and environmental conditions no longer operate simply as catalyzers, or confounding factors of the molecular causation of disease. Epigenetic measures of the effects of indicators such as socioeconomic position (SEP) provide a different understanding of biological differences and their role in disease risk stratification: These are sensitive organic traces of exposures and experiences [10]. As Cerutti and colleagues argued in this journal [10], different components of SEP (e.g., education, income, etc.) correspond to only partially overlapping biological signatures (i.e., specific DNA methylation differences). Of note, these differences are also affected by timing (i.e., there exist more or less sensitive periods over the life course), or duration (e.g., social mobility, cumulative effects) of exposures and fluctuate longitudinally depending on disease evolution patterns specific to each patient [11]. The variation in risk and outcomes of complex diseases is, in other words, *not* explained by biological or environmental factors taken in isolation: rather, this results from their combination, which produces a greater effect than the sum of their separate effects. On the one hand, this calls for multiplying epigenetic studies probing the associations between distinct social conditions, experiences, environmental exposures, and epigenetic differences [12]. On the other hand, this demands reconsidering notions of causality in these epigenetic associations which may be less linear than expected, and blur the boundary between genetic and environmental determinants of disease [13, 14].

The picture of disease etiology drawn by epigenetics is one of the looping effects between (material and social) environments and biology, past experiences and future predispositions, as well as nature and nurture in the production of disease. Elucidating these complex *biosocial loops* is the challenge epigenetics brings to the fore in the so-called post-genomic age*. Or, at least, this is the one it should be concerned with.*

## Why epigenetics is *not* a biosocial science

In fact, the development of epigenetic biomarkers of disease often pulls in a different direction. Specifically, one that departs from a complex and socially embedded view of biology. Let us again take the example of cardiovascular conditions. No doubt, epigenetic information can have an immediate impact on disease management. Epigenetic biomarkers are already at the forefront of clinical applications in precision cardiology [15]. These markers promise to contribute to patient diagnosis, prognosis, theragnosis, and therapy in several cardiovascular conditions—such as coronary artery disease, hypertrophic cardiomyopathy, acute myocardial infarction, and heart failure (Reviewed in [16, 17]). Although the additional benefits of using these biomarkers are still debated, epigenetic modifications find an increasing association with cardiovascular diseases and contribute to the quest for their early detection, risk prediction, and prevention [18, 19]. Of note, cardio-epigenetic biomarkers often do so by pointing to the mixed genetic and environmental etiology of these conditions: For instance, the combination of genetic and epigenetic information using machine learning has been shown to improve risk score classifiers and predictors for coronary heart disease [13]. But, what about the role of this kind of research in producing a full appreciation of the whole biological-and-social spectrum of factors shaping individual health?

Several social scientists have criticized how epigenetic research treats the social, environmental, and temporal modulators of disease (risk and etiology). Among these critical scholars, there is a fear of novel forms of reductionism in epigenetics that are no less worrying than those attributed to genetics. What if, critics ask, more than non-gene-centric biology, epigenetics turned out to be the science of a miniaturized and molecular version of the environment [20, 21]? Its insights and implications for an environmentally embedded view of health cannot be fully grasped if this science reduces biosocial loops to either methylation risk scores alone, or to “the effects [on human bodies] of proximate variables” [21, p. 17] such as socioeconomic status [10] or scores of traumatic stress [22]. We subscribe to a different (yet related) version of this criticism, which does not consider the reductionism of the life sciences problematic per se. This line of criticism rather asks whether the repertoire of tools and interventions of epigenetics can be expanded to complexify its grasp of biosocial processes of health differentiation. We acknowledge that available lab methods, statistical tools, populational measures, epidemiological questionnaires, and randomized clinical trials may not be suitable to fully dissect these biosocial loops. Furthermore, we are also aware of the clinical utility of ready-made, validated risk classifiers and predictors for the management of health conditions (such as cardiovascular diseases) [13]. Yet, we ask: are the existing methods and approaches in the field fated to overlook the thick social and cultural underpinnings of these conditions [23]? Is an approach that combines the scalability and predictive value of molecular tests with thicker stratifications through social markers amenable to experimentation in epigenetics? There might be a complexity in biosocial views of health that is incommensurable to the ordinary tools of the biomedical sciences. Yet, this does not mean the results of epigenetics ought necessarily to provide a poor picture of the environmental embeddedness of our health. Especially because the imbrication of these biological, psychological, social, and political factors represents an untapped potential for early prevention, risk prediction, and intervention [13].

While being general critiques of epigenetics, these concerns can be extended to cardiovascular epigenetics too. Cardiovascular epigenetic biomarkers certainly show promising healthcare applications [24], yet one could point to several elements that call for improved consideration of a biosocial perspective in their development. First, epigenetic studies of cardiovascular phenotypes are skewed in favor of an understanding of biomarkers as mere targets for molecular and, specifically, pharmacological intervention (epi-drugs). A growing literature points, in fact, to the pharmacological actionability of epigenetic differences, as in the reported case of the drug-tailoring based on genome-wide DNA methylation differences in hypertension [25]. A simple Web of Science search^2^ shows that therapeutic or pharmacological approaches to epigenomic signatures of cardiovascular diseases get considerably more attention than the actionability of these markers in primordial prevention. Plus, such preventive measures seem even more neglected when we consider that only a minority of the publications mentioning “prevention” does so without also including therapeutics as the potential application of epigenetic knowledge.^3^ While these indicative bibliometric measures cannot account for the nuances of a scientific debate, they suggest that cardio-epigenetic research emphasizes far less a socially embedded view of these risk factors than it focuses on pharmacological interventions to correct them.

Second, the understanding of the environment in this literature displays several limitations in light of a biosocial perspective on cardiovascular diseases. Some studies operationalize the environment just as light, temperature, and food [26]; others through proxy measures of social conditions such as socioeconomic status [10]. Some do underline the multiple pathways and loops between social interactions, psychological stress, and epigenetic predispositions to cardiovascular disease. Yet, they often do so only in animal experiments [27] or as part of conceptual discussions with little practical implementation [28, 29]. The integration of finer-grained measures of the environment and social conditions has found little translation in experimentation. Few studies exist that: (i) explore epigenetic mechanisms by which social influences (e.g., racialized inequalities in the USA) can become embodied health predispositions (e.g., racial disparity in cardiovascular risk and disease) [30]; (ii) probe the cumulative effect of social conditions, inequalities and exposures (e.g., pollutants, chemical hazards) in the (epigenetic) patterning of cardiovascular diseases in our societies [31]; (iii) explore the distinct associations and combinations of biological and proximal (e.g., lifestyle) or distal (e.g., social structures, environmental exposures) risk factors [13]. The lack of integration of these complex views of social–biological transitions in epigenome-wide association studies (EWAS) is a methodological gap that has found recognition only recently—including on this journal [10, 12]. Little consideration is given also, in the EWAS literature, to the need of differentiating the degrees of specificity, stability, and reversibility of epigenetic modifications (e.g., DNA methylation differences) in the face of clinical, behavioral, or social interventions [10, 11]. For instance, few studies have tried to dissect the age-specific associations between DNA methylation differences and cardiovascular phenotypes: The few results available suggest that epigenetic differences may be less relevant to predict cardiovascular outcomes in children than they are in adults [32]. In a nutshell, empirical research on social–biological loops producing epigenetic predispositions to cardiovascular diseases is limited. And, notably, this is due to the lack of fine-grained measures of the multiple sources, effects, temporalities, and mechanisms of the social exposures that produce these biological differences.

## Why that matters

This cursory look at research on the epigenetics of cardiovascular conditions hints at several gaps and challenges in the field. Roughly put, methods, approaches, and common research designs prevent epigenetics from fully embracing a biosocial understanding of these conditions. Yet, this piece should not be read as a damning diagnosis of failure. Neither its aim is revamping worn-out oppositions among disciplinary worldviews. As social scientist and clinical researcher joining forces and ideas around epigenetics, we want to emphasize the excitement this field offers to different scientific cultures and traditions of research. Epigenetics entertains a fascinating view of our biology as contingent on material and social environments, such as political, economic, and historical factors. It is a field where the research agendas of the life and social sciences—thought as irreconcilable over the last few decades—can converge and integrate [33]. What we decry, however, is a missing operationalization of these complex biosocial views of health into the experimental designs of the field. This is, we believe, a crucial point, which matters not only as untapped potential of a research field. Rather, failing to promote a biosocial perspective in epigenetics may hamper the pursuit of the objectives the field *has given itself*. If epigenetic mechanisms of complex diseases point to the deep socio-environmental embeddedness of our biology, why not study these modifications as something more than the extracellular environment’s effect on chemical modifications of DNA and chromatin? If socially patterned exposures and experiences connect with biological changes (and health outcomes) through multiple pathways and iterative loops, how could this complex view of health be put to test in populational studies? If biomedical and social sciences realize that the other’s approach is relevant, should integration and interdisciplinarity be made structurally part of projects and study designs in epigenetics? Finally, if social conditions are relevant for the social–biological patterning of health in epigenetics, should acting on these socio-environmental factors become a specific type of interventional study design in the field?

We hold a frugal theoretical stance on the practices of interdisciplinary, methodological, and interventional experimentation required to address these questions. Besides not being a damning diagnosis of failure, this piece is also not a call for a foundational endeavor leading to an alleged holistic biosocial science for the twenty-first century. We are fully aware that several organizational constraints (e.g., cost, project duration, etc.) could potentially hamper the development of such approaches in epigenetic research. Yet, our argument is meant as a critical consideration of the extent methods and typical study designs in epigenetics can and *should* interrogate the complexities of its research objects. As philosopher Georges Canguilhem [34] would put it, the biosocial loops behind complex diseases may be out of the reach of experimental sciences. However, these processes present epigenetic scientists with (what he would call) a “permanent exigency” to put “life in the living” (p. 62). According to Canguilhem, there is no science of living beings—nor any serious consideration of how health is patterned within a given milieu—without a fine-grained study of how these beings exist in and experience such a milieu. In other words, no biology of environmental effects is possible without a granular inquiry into how organisms inhabit—ecologically, relationally, socially, and materially—such environments. Much like the average epigenetic scientist reading this text, the French philosopher is aware that no easy method or ready-made protocol exists to study life in its milieu. This is the reason why he carefully employs the language of “exigency” toward experimental complexity—more than an idiom of theory-making—when discussing why researchers should engage with the environmental intricacies of their objects. Short of a foundational method of biosocial science lies, in other words, a practical *aspiration* toward the complexification of epigenetics—i.e., the pursuit of a more complex representation of the biosocial modulators of health in its designs, tools, and research objects. We believe that a multilayered assemblage of methodological experimentations can address the environmental and social embeddedness of our bodies in epigenetics. This patchwork of innovations could deliver a fertile complexification of epigenetics and its ways of studying the biological and social factors producing diseases. In the heterogeneity and idiosyncrasies of these practices lies the potential for epigenetics to become an integrative biosocial science. Genomics, some have diligently argued, “was a victim of its own premises and promises” [3, p. 86]. Will it be the same with epigenetics?

## Data Availability

Data sharing not applicable to this article as no datasets were exclusively generated for the current study.

## Footnotes

1 Population stratification designates the systematic differences between subpopulations in both allele frequencies and population phenotypes due to geographical and physical boundaries of these groups. Dynastic effects are the indirect influence that parental genotype has on offspring phenotype through expression in the parental phenotype. See (9) for a more detailed discussion.

2 We conducted in July 2021 a Web of Science search for the following string of terms: “ALL = (biomarker*) AND ALL = (epigenetic*) AND ALL = (cardio*).” This gave back a total of 710 papers, which constitute *bona fide* a literature base dealing with biomarkers for epigenetic differences in cardiovascular diseases. The search was then refined into two additional datasets where one term was added: one search included the word “ALL = (therap*),” the other “ALL = (prevent*).” This split the database of the first search into two sets: one containing publications that use any derivative of “therap*” – such as “therapy” or “therapeutic” and their plural forms (n = 282) – in relation to cardio-epigenetic biomarkers; one that includes any publication on the same topic making use of “prevent*” and any of its declensions “prevention,” “preventive,” “preventative” and their plural forms (n = 185).

3 We probed our two publication datasets for their overlap to assess whether therapeutic and preventive interventions are discussed in the same articles. Around 56% of the articles from the “ALL = (prevent*)” dataset was also part of the “ALL = (therap*) dataset. Thus, not only publications discussing therapeutic approaches to cardio-epigenetic biomarkers exceed those mentioning preventive measures in absolute (282 vs. 185). But the majority of articles examining preventive measures also included a mention of therapies and/or therapeutic approaches. While we cannot exclude that a fraction of these co-occurrences could be idiosyncratic (e.g., critical mentions of competing strategies of intervention in the epigenetics of cardiovascular diseases), these rough literature count and analysis show that, at least quantatitively, the debate internal to cardio-epigenetics is largely skewed in favor of pharmacological rather than social and structural interventions.
